# Fast and slow dynamics for classical and quantum walks on mean-field small world networks

**DOI:** 10.1038/s41598-019-55580-2

**Published:** 2019-12-16

**Authors:** Andre M. C. Souza, Roberto F. S. Andrade

**Affiliations:** 1 Departamento de Fisica, Universidade Federal de Sergipe, 49100-000 Sao Cristovao, SE Brazil; 2 Instituto de Física, Universidade Federal da Bahia, 40170-115 Salvador, BA Brazil; 3 Centre for Data and Knowledge Integration for Health (CIDACS), Instituto Gonçalo Muniz, Fundação Oswaldo Cruz (FIOCRUZ), 41745-715 Salvador, BA Brazil

**Keywords:** Mathematics and computing, Physics

## Abstract

This work investigates the dynamical properties of classical and quantum random walks on mean-field small-world (MFSW) networks in the continuous time version. The adopted formalism profits from the large number of exact mathematical properties of their adjacency and Laplacian matrices. Exact expressions for both transition probabilities in terms of Bessel functions are derived. Results are compared to numerical results obtained by working directly the Hamiltonian of the model. For the classical evolution, any infinitesimal amount of disorder causes an exponential decay to the asymptotic equilibrium state, in contrast to the polynomial behavior for the homogeneous case. The typical quantum oscillatory evolution has been characterized by local maxima. It indicates polynomial decay to equilibrium for any degree of disorder. The main finding of the work is the identification of a faster classical spreading as compared to the quantum counterpart. It stays in opposition to the well known diffusive and ballistic for, respectively, the classical and quantum spreading in the linear chain.

## Introduction

Quantum walks (QW)^1^ have great relevance for a large number of fundamental problems in physics, mathematics, optical devices, material properties in atomic and nano scales, and other natural sciences^2^.The increasing number of QW models have been mostly cast into two well characterized sets, which depend on whether evolving dynamics occur under the assumption of discrete (DTQW)^3–7^ or continuous time (CTQW)^8–12^. For CTQW’s, the unitary time evolution operator of probability transition between two quantum states is an exponential function of the Laplacian matrix representing the substrate. This approach is similar to the one used to describe continuous time transport by random walkers (CTRW) in classical non equilibrium statistical physics^9^.

The classical master-equation-type formalism, widely employed within the CTRW scheme^13^, can be extended to incorporate quantum-mechanical aspects. The resulting mathematical formulation, akin to that of tight-binding Hamiltonian models, reflects the similarity between time-evolution operators in statistical and in quantum mechanics. Within this analogy, CTQW stands as a linear problem, benefitting from many CTRW general results, as eigenvalue and eigenvector properties. Like CTRW, many QW models now describe transport properties in diverse substrates, which simply requires writing the local transition probability in terms of the proper adjacency matrix of complex networks.

In spite of similar algebraic structures, the time evolution of CTQW and CTRW indicate vastly different physical properties, no matter whether the system is defined on regular or complex substrates. For instance, the faster ballistic spreading of CTQW as compared to the CTRW’s classical diffusive behavior, the fact that, in the absence of traps, CTQW’s are time-inversion symmetric and no energy equipartition takes place at long times. Further, the quantum system keeps memory of the initial conditions, as evidenced by the occurrence of quasi-revivals^14^. We also remark that CTQW models coherent exciton transport on a connected network, replacing the system’s Hamiltonian by the Laplace matrix^15^.

A large number of works have reported properties of both DTQW and CTQW on complex networks, including geometrically defined structures, like the Apollonian network^16,17^, or complex networks with different degrees of randomness, e.g., Erdös-Rényi, Watts-Strogatz small-world, Barabasi-Albert scale-free, dendrimer, or polymer^14,18–26^. In this work we investigate the dynamical properties of CTQW and CTRW on the so-called mean-field small-world (MFSW) networks^26^. They have been recently explored in several studies of physical models on networks using analytical approaches. A main reason supporting this choice is the fact that, once they are represented by circulant adjacency matrices, several exact properties of their eigenvalue spectra are known. Here, we advance far beyond the basic ideas developed in^26^ to derive exact analytical results for the transition probabilities. The reliability of our approach is illustrated through the comparison with numerical results obtained by working directly the Hamiltonian of the model.

The two-parametric (*k*, *q*) family of MFSM networks with *N* nodes is defined by a weighted adjacency matrix^26^ with the circulant property, which must have the following structure^27^:

1 $${\hat{A}}_{MF}=[\begin{array}{lllll}0 & {c}_{1} & {c}_{2} & \cdots  & {c}_{N-1}\\ {c}_{N-1} & 0 & {c}_{1} & \cdots  & \vdots \\ \vdots  & \vdots  & \vdots  & \ddots  & \vdots \\ {c}_{2} & {c}_{3} & {c}_{4} & \cdots  & {c}_{1}\\ {c}_{1} & {c}_{2} & {c}_{3} & \cdots  & 0\end{array}].$$

Since the network is assumed to be undirected, the elements *c*_*l*_ occupying the successive diagonals also satisfy *c*_*l*_ = *c*_*N*−*l*_. The elements *c*_*l*_ of a MFSW network can assume assume only two different values, so that the connections must belong to one of two subsets denoted by *S*_1_ and *S*_2_. The two possible values of *c*_*l*_ are defined in terms of two parameters *k* and *q* according to

2 $${c}_{l}=\{\begin{array}{ll}1-q(1-w) & \,{\rm{if}}\,l\,\in \,{S}_{1},\\ w & \,{\rm{if}}\,l\,\in \,{S}_{2},\end{array}$$

where

3 $$w=\frac{qk}{N-1-(1-q)k}.$$

**Figure 1:**
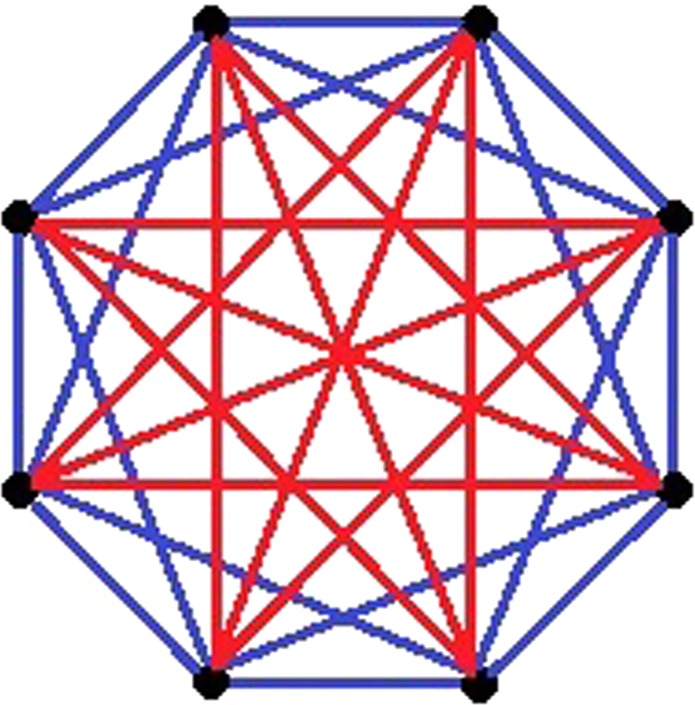
Illustration of a small-world mean-field network, for *N* = 8, *k* = 4 and *q* = 0.1. Blue and red bounds correspond, respectively, to elements of *S*_1_, for which *c*_*l*_ = −31/34, and of *S*_2_, for which *c*_*l*_ = −4/34.

The connections in *S*_1_ correspond to the elements *c*_*l*_ and *c*_*N*−*l*_, with labels *l* = 1,2, ..., *k*/2;...; *N* − *k*/2, ..., *N* − 1, while those in set *S*_2_ are associated to labels *l* = 1 + *k*/2, ..., *N* − 1 − *k*/2. *k* ∈ [2,*N* − 1] and *q* ∈ [0,1] represent, respectively, the average node degree and topological randomness as compared to a closed chain where each node is connected to its closest *k* neighbors. The condition *k* = 2, *q* = 0 corresponds to the usual nearest neighbor (NN) circular chain. The network topology resulting from this definition can be exemplary visualized in Fig. 1, for the case *N* = 8, *k* = 4 and *q* = 0.1. It clearly shows that each site of the network receives links from its *k*/2 nearest neighbors on both sides. The extreme values of *q* = 0 and 1 correspond, respectively, to a uniform circle graph with *k* neighbors and a homogeneous complete graph, while intermediate values of *q* are fully connected networks with two distinct weights. The conditions for a small-world network are obtained by small non-zero *q* values^26^. For the sake of simplicity in deriving some analytical expressions, in this paper we assume that *N* and *k* are even integers.

## Results

### Adjacency matrix spectrum

The matrix ${\hat{A}}_{MF}$ also represents a tight-binding Hamiltonian for a quantum particle system, for which the CTQW dynamics is described by the Laplacian matrix

4 $$\hat{L}={c}_{o}\hat{I}-{\hat{A}}_{MF},$$

where *c*_*o*_ = *k* and $\hat{I}$ represents the *N* × *N* identity matrix.

It is well known^27^ that the eigenvalues and eigenvectors of any circulant matrix of order *N* can be written, respectively, in terms of the following analytical expressions

5 $${\Lambda }_{l}=\mathop{\sum }\limits_{j=0}^{N-1}\,{c}_{j}{e}^{-i\frac{2\pi jl}{N}}$$

and

6 $$|{\alpha }_{l}\rangle =\frac{1}{\sqrt{N}}{[1,{e}^{-2\pi i\frac{l}{N}},{e}^{-2\pi i\frac{2l}{N}},\cdots ,{e}^{-2\pi i\frac{(N-1)l}{N}}]}^{T}.$$

From Eqs. (2) and (4) it is easy to see that the ground state eigenvalue and eigenvector are, respectively, ${\Lambda }_{0}=\sum _{j}{c}_{j}=0$ and $|{\alpha }_{0}\rangle =\frac{1}{\sqrt{N}}{[1,1,1,\cdots ,1]}^{T}$, a general property of Laplacian matrices. Furthermore, using Λ_0_ = 0, *c*_*l*_ = *c*_*N*−*l*_, and substituting Eq. (2) into Eq. (5) leads to

7 $${\Lambda }_{l}(k,q,N)=(1-q)(1-w)({R}_{k}^{0}-{R}_{k}^{l})+wN(1-{\delta }_{l,0}),$$

where *δ*_*l*,0_ is the Kronecker’s delta and

8 $${R}_{k}^{l}\equiv 2\mathop{\sum }\limits_{n=1}^{k/2}\,\cos (\frac{2n\pi l}{N})=\frac{\sin (\frac{(k+1)\pi l}{N})}{\sin (\frac{\pi l}{N})}-1.$$

In the thermodynamic limit *N* → ∞, using that lim_*N*→∞_(1 − *w*) = 1 and lim_*N*→∞_*wN* = *kq*, from Eqs. (7) and (8) we obtain

9 $${\Lambda }_{l}(k,q,N\to \infty )=k(1-q{\delta }_{l,0})-(1-q){R}_{k}^{l}.$$

As we are considering *N* and *k* even, we have *R*_*k*_^*N*−*l*^ = *R*_*k*_^*l*^ and Λ_*N*−*l*_ = Λ_*l*_ for *l* = 1, 2, ..., *N*/2 − 1. With the exception of the non-degenerated levels Λ_0_ = 0 and Λ_*N*/2_, all eigenvalues are double degenerated.

### Analytical results for the transition probabilities

The CTRW dynamics on a network is described by the probability *P* of finding the w of time, which obeys the equation ∂*P*/∂*t* = −$\hat{L}P$. Within the CTQW framework, the time evolution of a quantum particle is described by the operator $\hat{U}(t)={e}^{-i\hat{L}t/\hslash }$ that acts on the state vector |Ψ(*j*, *t*)〉, with position *j* and time *t*. We set $\hslash$ = 1 and assume the particle starting at time *t* = 0 on a position *j*_0_ of the network, that corresponds to state |Ψ(*j*, *t*)〉 = |*j*_0_〉 = *δ*_*j*,*j*0_. The state |*j*_0_〉 represents one of the states of the set |1〉 = [1, 0, 0, ..., 0], |2〉 = [0, 1, 0, ..., 0], …, |*m*〉 = [0, 0, ..., 1, ..., 0],…, |*N*〉 = [0, 0, 0, ..., 1], that form a complete, ortho-normalized basis of the Hilbert space. From Eq. (6), it is easy to see that

10 $$\langle m|{\alpha }_{l}\rangle =\frac{1}{\sqrt{N}}{e}^{-2\pi il(m-1)/N}\,m=1,2,\mathrm{..}.,N.$$

The classical and quantum transition probabilities between two states (thinking as nodes on a network) are, respectively,

11 $${P}_{mj}(t)=\langle m|{e}^{-t\hat{L}}|j\rangle $$

and

12 $${\Pi }_{mj}(t)={|\langle m|{e}^{-it\hat{L}}|j\rangle |}^{2}.$$

Using Eqs. (6) and (10) into Eqs. (11) and (12), we obtain

13 $${P}_{mj}(t)=\frac{1}{N}\mathop{\sum }\limits_{l=0}^{N-1}\,{e}^{-t{\Lambda }_{l}-\frac{2\pi }{N}i(m-j)l}$$

and

14 $${\Pi }_{mj}(t)={|\frac{1}{N}\mathop{\sum }\limits_{l=0}^{N-1}{e}^{-i(t{\Lambda }_{l}-\frac{2\pi }{N}(m-j)l)}|}^{2}\,.$$

Both general equations for the classical (13) and quantum (14) cases can be expanded in terms of real exponential and cosine functions, from which asymptotic expressions can be obtained in terms of Bessel functions. Therefore, after inserting Eq. (7) into Eq. (13) we obtain, for the classical probabilities

15 $${P}_{mj}(t)=\frac{1+{e}^{-t{C}_{0}}}{N}+\frac{1}{N}\mathop{\sum }\limits_{l=1}^{\frac{N}{2}-1}\,{e}^{-t{C}_{l}}\,\cos (\frac{2\pi }{N}(j-m)l)$$

where *C*_0_ = (1 − *q*)(1 − *w*)[*k* + 1 − (−1)^(*k*/2)^] + *wN* and *C*_*l*_ = (1 − *q*)(1 − *w*)(*R*_*k*_^0^ − *R*_*k*_^*l*^) + *wN*.

On the other hand, in the quantum cases it is easy to see, from Eq. (14), that

16 $${\Pi }_{mj}(t)=\frac{1}{{N}^{2}}\mathop{\sum }\limits_{l=0}^{N-1}\,\mathop{\sum }\limits_{p=0}^{N-1}\,\cos \,[{\theta }_{mj}^{l}(t)-{\theta }_{mj}^{p}(t)],$$

where *θ*_*mj*_^*l*^(*t*) = *t*Λ_*l*_ + 2*πl*(*j* − *m*)/*N*. We can observe that in both the classical and quantum cases the transitions depend only on the difference | *j* − *m*|.

The limit *q* = 1 is trivial and has already been explored in the literature^15,19^. It is the network in which every sites are linked with the same hopping energy. It is easy to see that

17 $${P}_{mj}(t)={\delta }_{mj}{e}^{-t\frac{kN}{N-1}}+\frac{1}{N}(1-{e}^{-t\frac{kN}{N-1}})$$

and

18 $${\Pi }_{mj}(t)=\frac{1}{N}+\frac{2}{{N}^{2}}\mathop{\sum }\limits_{l=1}^{N-1}\,\cos [\frac{kNt}{N-1}+\frac{2\pi }{N}l(j-m)]+{D}_{mj},$$

where ${D}_{mj}=\mathop{\sum }\limits_{l=1,p=1;l\ne p}^{N-1}\,\cos [\frac{2\pi }{N}(l-p)(j-m)]$. For transitions between the same site

19 $${\Pi }_{mm}(t)=\frac{{N}^{2}-2N+2}{{N}^{2}}+\frac{2(N-1)}{{N}^{2}}\,\cos (\frac{kNt}{N-1}).$$

In the next section we analyse the case 0 ≤ *q* < 1. We illustrate properties of classical and quantum dynamical behavior based on the evaluation of Eqs. (15) and (16). These equations are start points for the derivation of asymptotic expressions in terms of Bessel function.

### Classical dynamics

**Figure 2:**
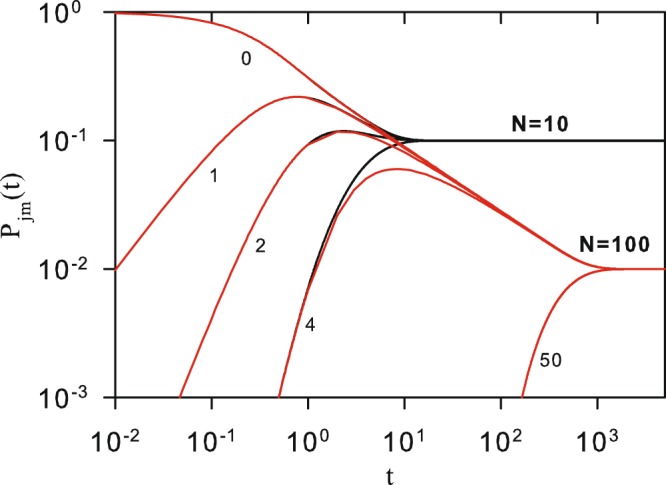
Time evolution of the classical transition probability *P*_*mj*_(*t*) for *q* = 0. Black (red) curves corresponds to *N* = 10 (*N* = 100) for |*m* − *j*| = 0, 1, 2, 4 and 50 (only for the case *N* = 100). Curves are analytical results from Eq. (15).

Figure 2 shows *P*_*mj*_(*t*) as a function of *t*, for *q* = 0, *N* = 10 and 100, and different values of |*m* − *j*|. For small *t*, the probability curves for different *N* overlap almost exactly indicating no significant difference. For *t* → ∞, the result ${\mathrm{lim}}_{t\to \infty }{P}_{mj}(t)=\frac{1}{N}$ indicates equal probability 1/*N* for every site transition at long time. This asymptotic result is valid for any *q*, as a result of Eq. (15) and from the fact that *C*_*l*_ > 0. Figure 2 indicates that, for a fixed *N*, the asymptotic regime is reached at approximately the same time for any value of |*m* − *j*|. Thus, we can define the classical equilibrium time *t*_*ec*_, at which every *P*_*mj*_(*t*_*ec*_) converges to 1/*N*, from the expression for *P*_*jj*_(*t*_*ec*_). Namely, the equilibrium time *t*_*ec*_(*N*) is defined as the minimum value of *t* that satisfies the condition

20 $$|\frac{{P}_{jj}({t}_{ec})-\frac{1}{N}}{\frac{1}{N}}|\le \epsilon ,$$

where $\epsilon$ is a small dimensionless constant.

For *q* = 0, an analytical estimation for *t*_*ec*_(*N*) in the limit *N* ≫ 1 can be derived. As in this situation, 0 = Λ_0_ < Λ_1_ = Λ_*N*−1_ ≪ Λ_*l*_, *l* ∈ [2,*N* − 2], all but the three dominant terms in Eq.(13) can be neglected. After some algebraic manipulations with Eq. (7), we obtain Λ_1_(2, 0, *N*) ≈ 4(*π*/*N*)^2^, Λ_1_(4, 0, *N*) ≈ 20(*π*/*N*)^2^, …, Λ_1_(*k*, 0, *N*) ≈ *A*_*k*_(*π*/*N*)^2^, where *A*_*k*_ is successively defined by *A*_*k*+2_ = *A*_*k*_ + *k*^2^. This leads to the result

21 $${t}_{ec}(N)\approx \frac{{N}^{2}}{{A}_{k}}.$$

**Figure 3:**
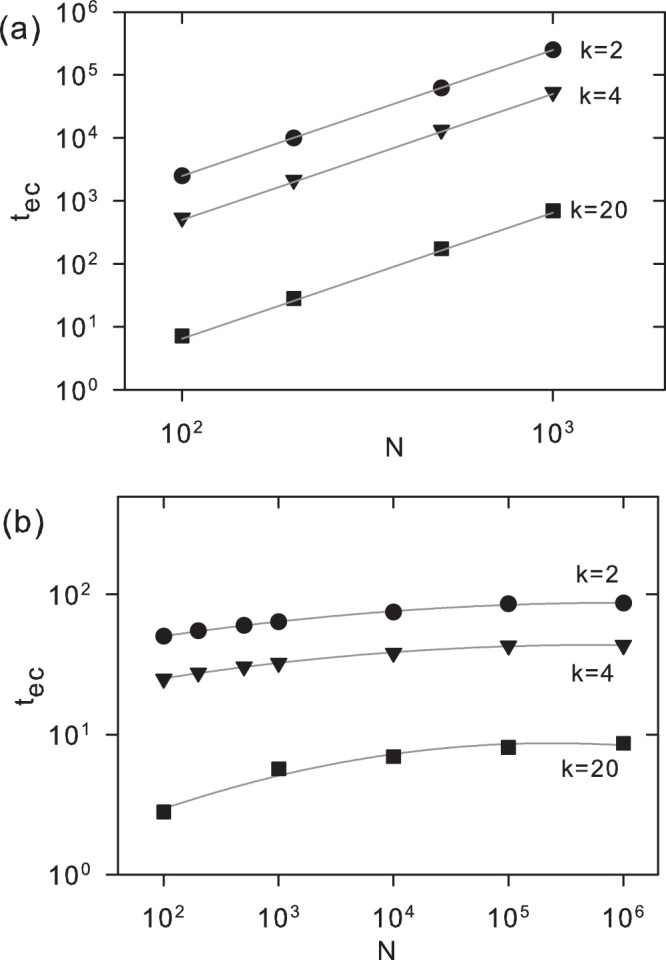
Equilibrium time *t*_*ec*_(*N*) defined by Eq. (20) as a function of *N*, for $\epsilon$ = 2*e*^−*π*2^ ≅ 0.0001034. (**a**) *q* = 0 and *k* = 2, 4 and 20. Symbols and curves represent, respectively, numerical results from Eq. (15) and analytical approximation in Eq. (21). (**b**) *q* = 0.1 and *k* = 2, 4 and 20. Symbols represent numerical result from Eq. (15), while curves are eye guides.

Figure 3(a) shows numerical and analytical results for *t*_*ec*_(*N*) as a function of *N* when *q* = 0, for *k* = 2, 4 and 20. The analytical estimation for *t*_*ec*_(*N*) given by Eq. (20) is consistent with the numerical results, indicating a power law divergence with exponent 2. As we can not neglect the eigenvalues Λ_*l*_ (*l* > 1) when *q* > 0, it has not been possible to derive a general analytic expression valid for all *q*. Figure 3(b) presents *t*_*ec*_ versus *N* when *q* = 0.1, *k* = 2, 4 and 20, where the numerical values were obtained from Eq. (15). We observe that *t*_*ec*_(*N*) is several orders of magnitude smaller than for the case *q* = 0, a feature that is also valid for the whole interval 0 < *q* < 1. Thus, *t*_*ec*_(*N*) no longer follows a power law dependence with respect to *N*, but converges exponentially to a finite value. The results indicate that the asymptotic value for *t*_*ec*_(*N*) in the limit *N* → ∞ is *t*_*ec*_ ≈ (*kq*)^−1^.

**Figure 4:**
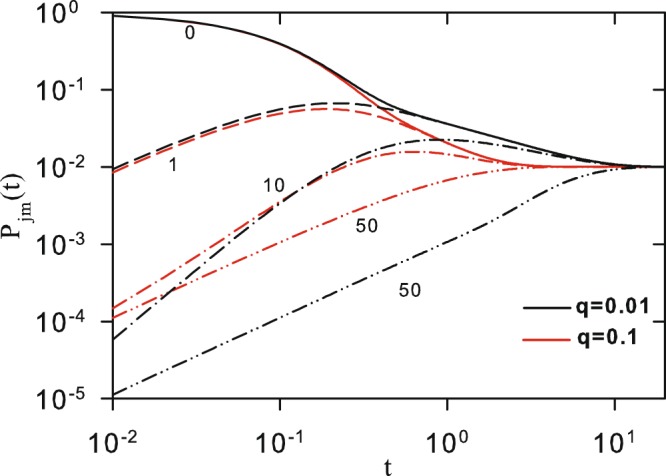
Time evolution of the classical transition probability *P*_*mj*_(*t*) for *N* = 100. Black (red) curves corresponds to *q* = 0.1 (*q* = 0.01) for |*m* − *j*| = 0, 1, 10 and 50. Values of |*m* − *j*| are localized close the curves. Curves are analytical results from Eq. (15).

**Figure 5:**
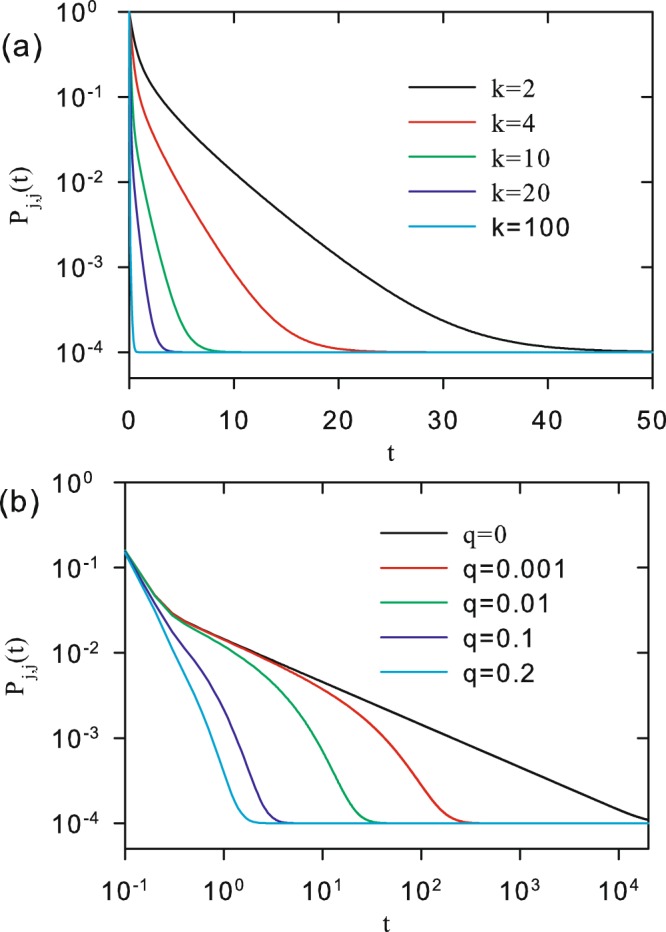
Time evolution of the classical transition probability *P*_*mj*_(*t*) for *N* = 10000. (**a**) *q* = 0.1 for *k* = 2, 4, 10, 20 and 100. (**b**) *k* = 20 for *q* = 0, 0.001, 0.01, 0.1 and 0.2. Curves are analytical results from Eq. (15).

Figure 4 shows *P*_*mj*_(*t*) versus *t*, for *N* = 100, *q* = 0.01 and 0.1, and different values of |*m* − *j*|. It shows that, for a fixed *N*, the convergence of *P*_*mj*_(*t*) to = 1/*N* is faster when *q* increases. The behavior for *P*_*j*,*j*_(*t*) as a function of *t* is shown in Fig. 5 for *N* = 10000. In panels (a) and (b) we show, respectively, curves for different values of *k* and constant *q*, and different values of *q* and constant *q*. We see that the higher the values of *k* and/or *q*, the faster is the decay of *P*_*j*,*j*_(*t*) to its equilibrium value. Such overall behavior is somewhat expected, once the number of connections in the network increases with *k*, while the energy for jumps between sites of the network decreases when *q* increases.

In the limit *N* → ∞, this behavior can be explained by the following steps. First insert Eq. (9) into Eq. (13), and note that^28^

22 $${e}^{t\cos (x)}=\mathop{\sum }\limits_{n=-\infty }^{\infty }\,{I}_{n}(t){e}^{inx},$$

where *I*_*n*_(*t*) is the Modified Bessel function. Next, after straightforward calculations, we obtain

23 $${P}_{mj}(t)={e}^{-kt}\sum _{{n}_{1}}\,\mathrm{..}.\sum _{{n}_{k}}\,{I}_{{n}_{1}}(\tilde{t})\mathrm{..}.{I}_{{n}_{k}}(\tilde{t}){\delta }_{{n}_{1}+2{n}_{2}+\mathrm{..}.+\frac{k}{2}{n}_{k},j-m}$$

where $\tilde{t}=2(1-q)t$. From the above expression, we can easily re-obtain the results to one-dimensional nearest-neighbor chain (*q* = 0 and *k* = 2)

24 $${P}_{mj}(t)={e}^{-2t}\times \{\begin{array}{ll}{I}_{0}(2t), & {\rm{for}}\,m=j,\\ 2{I}_{|m-j|}(2t), & {\rm{for}}\,m\ne j.\end{array}$$

For *t* ≫ 1, it is possible to use the asymptotic limit of the Modified Bessel function for large *t* ${I}_{n}(t)\approx {e}^{t}/\sqrt{2\pi t}$^28^. Two different situations, which have been discussed before, emerge from the expression in (23): when (i) *q* = 0, a polynomial decay ${P}_{jj}(t)\approx {t}^{-\frac{1}{2}}$ is observed for all *k*; (ii) for *q* ≠ 0, the behavior changes sharply into an exponential decay *P*_*jj*_(*t*) ≈ *e*^−*kqt*^. Thus, we see that any infinitesimal disorder is sufficient to completely change the approach to the asymptotic regime.

To study the diffusion of the classical particle in the system, we can also define $\langle x(t)\rangle =\sum _{j}\,j{P}_{mj}(t)$ and $\langle {x}^{2}(t)\rangle =\sum _{j}\,{j}^{2}{P}_{mj}(t)$, which are independent of *m*. From Eq. (23), we obtain that

25 $$\langle x(t)\rangle =0,\,\langle x(t){x}^{2}(t)\rangle ={A}_{k}(1-q)t{e}^{-kqt}.$$

The last expressions shows again a sharp transition from a normal diffusion at *q* = 0 to an exponential sub-diffusive behavior for *q* > 0.

### Quantum dynamics

**Figure 6:**
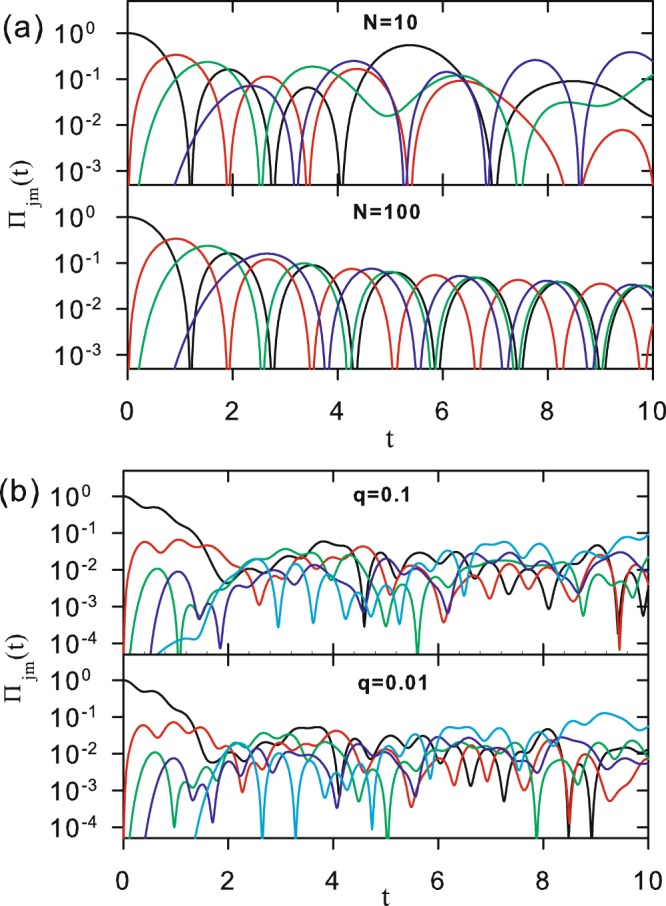
Time evolution of the quantum transition probability Π_*mj*_(*t*) for (**a**) *q* = 0 and *k* = 2 for *N* = 10 (upper panel) and *N* = 100 (lower panel). Curves represent: |*m* − *j*| = 0 (black), 1 (red), 2 (green), 4 (blue), and 50 (cian) (only for the case *N* = 100). (**b**) *N* = 100 and *k* = 20 for *q* = 0.1 (upper panel) and *q* = 0.01(lower panel). Curves are analytical results from Eq. (16), corresponding to |*m* − *j*| = 0 (black), 1 (red), 10 (green), and 50 (blue).

**Figure 7:**
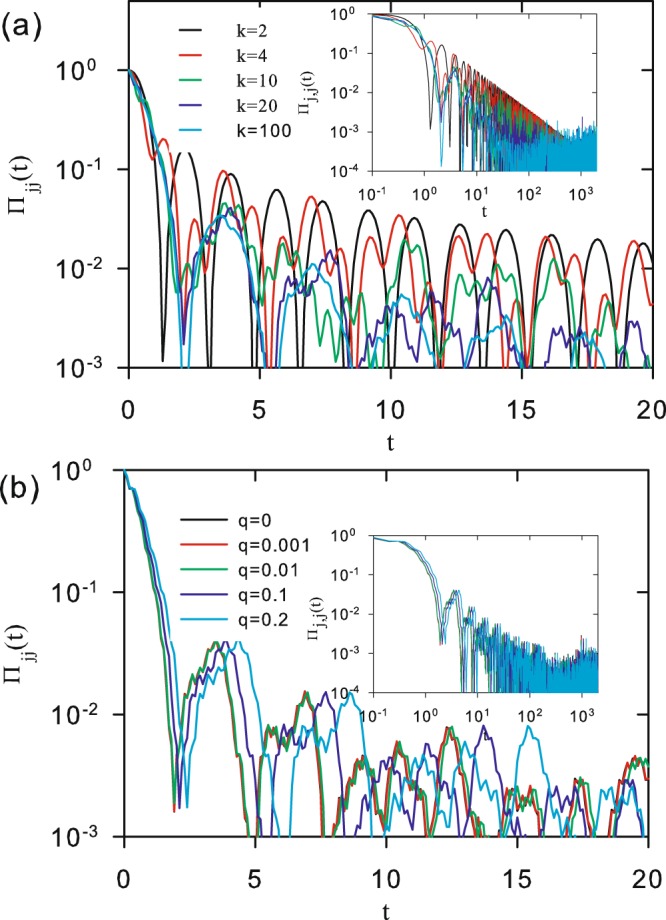
Time evolution of the quantum transition probability Π_*mj*_(*t*) for *N* = 10000. (**a**) *q* = 0.1 for *k* = 2 (black), 4 (red), 10 (green), 20 (blue), and 100 (cian). (**b**) *k* = 20 for *q* = 0 (black), 0.001 (red), 0.01 (green), 0.1 (blue), and 0.2 (cian). Logarithmic time axis in both insets highlights the asymptotic behavior. Curves correspond to analytical results in Eq. (16).

Figure 6(a) shows Π_*mj*_(*t*) versus *t* for *q* = 0 and the same parameter values of *N* and |*m* − *j*| used in Fig. 2. Unlike the classical behavior, characterized by an asymptotic value for the transition probability, a clear oscillatory pattern for Π_*mj*_(*t*) is the signature of the quantum case. It is always present, either when *q* > 0, as shown in Fig. 6(b), or for much larger values of *N*. In this case, the decay of the oscillation amplitudes, which can already been identified for small systems, becomes quite evident. This is made evident in Fig. 7, for both the short and asymptotic time regimes, when the case *N* = 10^4^ is considered.

**Figure 8:**
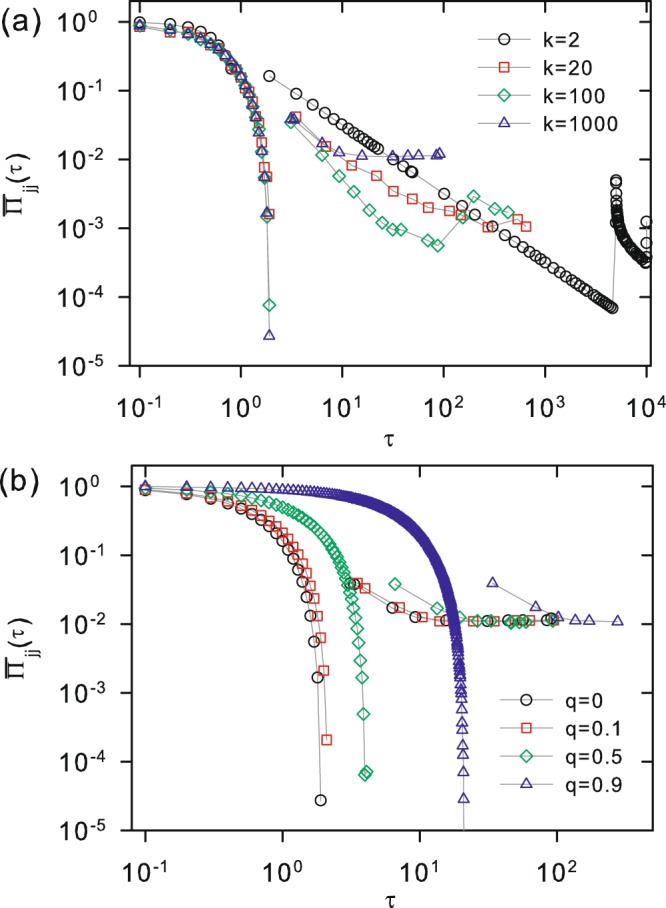
Local maxima of quantum transition probability ${\bar{\Pi }}_{jj}(\tau )$ as a function of *τ* for *N* = 10000. In (**a**) *q* = 0 is fixed, while *k* = 2 (black circles), 20 (red squares), 100 (green diamonds) and 1000 (blue triangles). In (**b**) *k* = 1000 is fixed, while *q* = 0 (black circles), 0.1 (red squares), 0.5 (green diamonds) and 0.9 (blue triangles).

In order to measure the time decay of the transition amplitudes, we generate two discrete series {${\bar{\Pi }}_{jj}(t)\}$ and {*τ*}, the values of which are defined the local maxima of Π_*jj*_(*t*), and the corresponding values of *t* where they occur. By removing the fluctuating part, we can work with a very small fraction of all points shown in Figs. 6 and 7. The behavior of the discrete set of selected points is illustrated in Fig. 8 for different combinations of *k* and *q*. It shows that three distinct decay regimes can be identified. Indeed, for small times ($0 < t\lesssim 1\equiv {t}_{x1}$), ${\bar{\Pi }}_{jj}(\tau )$ is first characterized by a fast transient exponential decay, at the end of which it becomes vanishingly small. This defines the first crossover time *t*_*x*1_, which depends on the values of *q* and *k*. Then, a sharp transition occurs, in which ${\bar{\Pi }}_{jj}(\tau )$ recovers some 5–10% of it’s initial value, marking the starting point of a second decay regime. It encompasses intermediate time interval ${t}_{x1}\lesssim t\lesssim {t}_{x2}$, when ${\bar{\Pi }}_{jj}(\tau )$ has a polynomial decay. As before, *t*_*x*2_ also depends on *q* and *k*. Finally, in the interval ${t}_{x2}\lesssim t$, the maxima amplitude enters a third and last regime, in which it has roughly stabilized behavior. Therefore, it is legitimate to identify *t*_*x*2_ with the quantum equilibrium time *t*_*eq*_, as it plays the same role of *t*_*ec*_ defined by Eq. (20) for the classical case.

Approximate analytical estimations for *t*_*eq*_, as well as for the average value about which Π_*jj*_(*t*) fluctuates for *t* > *t*_*eq*_, are much more complex to be obtained as compared to the classical case. Nevertheless, a few steps towards this goal can be done. Taking into account that the short time contribution is averaged out in the *t* → ∞ limit, let us first define

26 $${\chi }_{mj}=\mathop{\mathrm{lim}}\limits_{T\to \infty }\frac{1}{T}{\int }_{0}^{T}\,{\Pi }_{mj}(t)dt.$$

After inserting Eq. (16) into Eq. (26), and performing some lengthy but straightforward calculations, it is possible to obtain

27 $${\chi }_{mj}(k,q,N)=\frac{1}{{N}^{2}}\mathop{\sum }\limits_{l=0}^{N-1}\,\mathop{\sum }\limits_{p=0}^{N-1}\,\cos [\frac{2\pi (l-p)(j-m)}{N}]{\delta }_{{\Lambda }_{l},{\Lambda }_{p}}.$$

From the definition of Λ_*l*_ given by Eq. (7), we observe that *δ*_Λ*l*,Λ*p*_ is independent of *q* (for *q* ≠ 1). Because of this, we can simplify the above notations, i.e., *χ*_*mj*_(*k*,*q*,*N*) = *χ*_*mj*_(*k*,*N*). Therefore, for any *q* < 1, in the case *m* = *j* we can write

28 $${\chi }_{jj}(k,N)=\frac{1}{{N}^{2}}\mathop{\sum }\limits_{l=0}^{N-1}\,\mathop{\sum }\limits_{p=0}^{N-1}\,{\delta }_{{\Lambda }_{l},{\Lambda }_{p}}.$$

For *k* = 2, which corresponds to the one-dimensional NN circular chain, we can use Eq. (7) to write the following equation for the eigenvalue difference

29 $${\Lambda }_{l}-{\Lambda }_{p}=4\,\sin [\frac{\pi }{N}(l-p)]\sin [\frac{\pi }{N}(l+p)].$$

We note that the first sine function has *N* zeros, whenever *l* = *p*. In addition, the second function has *N* − 2 zeros, when the condition *l* + *p* = *N* is satisfied. Hence,

30 $${\chi }_{jj}(2,q,N)=\frac{2}{N}-\frac{2}{{N}^{2}}.$$

We understand that a similar expression for the general case *j* ≠ *m*, as well for other values of *k*, can hardly be obtained due the complexity of the involved expressions. For instance, even in the case *k* = 2 this would require count all cases for which $\cos [\frac{2\pi (l-p)(j-m)}{N}]\ne 0$.

Using a similar approach to that in the previous subsection, let us consider the expression (see^28^)

31 $${e}^{it\cos (x)}=\mathop{\sum }\limits_{n=-\infty }^{\infty }\,{J}_{n}(t){e}^{in(x+\frac{\pi }{2})}$$

where *J*_*n*_(*t*) denotes the Bessel function. Proceeding along the same lines to the classical case, we find that in the *N* → ∞ limit, the corresponding quantum expression becomes

32 $${\Pi }_{mj}(t)={[\sum _{{n}_{1}}\mathrm{..}.\sum _{{n}_{k}}{J}_{{n}_{1}}(\tilde{t})\mathrm{..}.{J}_{{n}_{k}}(\tilde{t}){\delta }_{{n}_{1}+2{n}_{2}+\mathrm{..}.+\frac{k}{2}{n}_{k},j-m}]}^{2}$$

For the one-dimensional NN chain (*k* = 2), we obtain again

33 $${\Pi }_{mj}(t)=\{\begin{array}{ll}{[{J}_{0}(t)]}^{2}, & {\rm{for}}\,m=j,\\ 2{[{J}_{|m-j|}(t)]}^{2}, & {\rm{for}}\,m\ne j.\end{array}$$

For large *t*, *J*_*n*_(*t*) ≈ *t*^−1/2 ^^28^, with the consequence that Π_*mj*_(*t*) ≈ *t*^−1^ for any *k* and *q*. This also leads to the asymptotic behavior *χ*_*mj*_ → 0.

Finally, the analysis of the quantum diffusion based on $\overline{x(t)}=\sum _{j}\,j{\Pi }_{mj}(t)$ and $\overline{{x}^{2}(t)}=\sum _{j}\,{j}^{2}{\Pi }_{mj}(t)$, independent of *m*, leads to the results

34 $$\overline{x(t)}=0\,{\rm{and}}\,\overline{{x}^{2}(t)}\approx {t}^{2},$$

which corresponds to the expected ballistic diffusion. This result is valid for any value of *k*.

**Figure 9:**
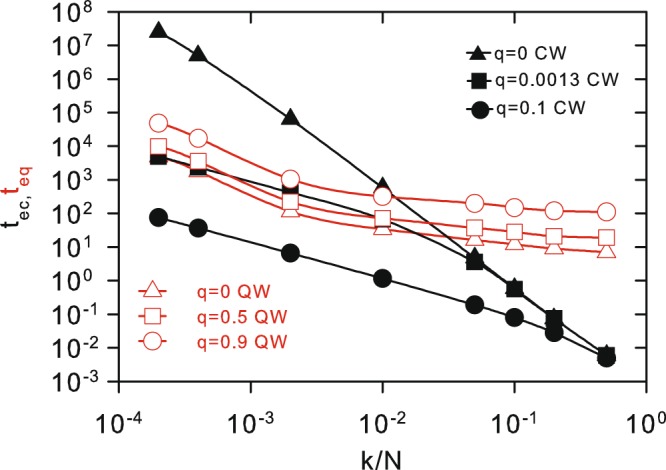
Quantum and classical equilibrium time (*t*_*eq*_ and *t*_*ec*_) as a function of *k*/*N* when *N* = 10^4^ for corresponding QW and CW walkers. Results for QW’s when *q* = 0, *q* = 0.5 and *q* = 0.9. Results for CW’s when *q* = 0, *q* = 0.0013 and *q* = 0.1 are also included, using the same parameter values for *t*_*ec*_ as in Fig. 3.

Figure 9 shows numerical results for the dependence of *t*_*ec*_ and *t*_*eq*_ with respect to *k*/*N*. Different values of *q* for CTRW and CTQW are considered The important aspect shown in the graph is that, irrespective of exponential or power law convergence to the equilibrium value, the classical spreading becomes more rapid on MFSW for any value of *q* and sufficiently large *k*. Thus, it reverts a well-known behavior resulting from the comparison between CTQW and CTRW spreading times, first obtained for the simple *k* = 2 circle chain. Since this conclusion follows from an exact analytical approach, it uncovers one more interesting property of CTQW.

## Discussion

This work presented a comprehensive analysis of quantum walks on MFSW networks. To achieve this goal, it systematically explored mathematical properties of the eigenvalue spectra of circulant matrices. Analytical expressions for the walker transition probability were derived for both the classical and quantum cases, which have been expressed in terms of modified and standard (*I*_*n*_(*t*) and *J*_*n*_(*t*)) Bessel functions respectively. As expected, for any finite substrate with *N* sites, both transition probabilities converge to an asymptotic equilibrium value. Here we remind that, although the oscillatory behavior never settles down for the CTQW, other measures like the average value over suitable time period or largest maxima over the same interval can be taken as a indicative that an equilibrium state has been reached.

A most amazing result from our analysis follows when they are compared with the scenario for continuous time models on the linear chain with limited number of neighbors: there, well characterized diffusive and ballistic spreadings indicate that classical dynamics proceeds at a slower pace than the quantum dynamics. Here, however, our results indicate the opposite scenario: the behavior of the classical and quantum transition probability as a function of time indicate, respectively, exponential and power law decay to the equilibrium value. Only when the disorder parameter *q* vanishes a typical power law behavior characteristic for the linear chain is recovered.

We finally comment that, as in MFSW networks all sites are interconnected even in the limit of infinitesimal but non-zero disorder, it does not allow to a satisfactorily tractable analysis of DTQW model. For instance, in such case, the coin operator would have to be represented by a high rank matrix, e.g, the *N* × *N* Fourier operator. Nevertheless, the reversion of the slow and fast dynamics identified for the continuous time regime hints that unexpected results may also be found in DTQW models.

## Methods

All analytical results for the classical and quantum transition probabilities were derived with classical mathematical methods with widespread use in quantum walks^2,14^. These include Fourier transforms, general matrix algebra, general properties of the eigenvalue spectrum of circulant matrices, and asymptotic properties of Bessel functions.

The numerical results were obtained from the evaluation of the time dependent transition probability. The numerical values of the exact analytical expressions were obtained by codes written by the authors in FORTRAN language.
